# A Study of the Effect of Aniline Curing Agent Bridge Bonding Groups on Charge Injection at the Copper/Epoxy Interface

**DOI:** 10.3390/ma18214951

**Published:** 2025-10-30

**Authors:** Liuhuo Wang, Sukai Hu, Zhiwu Xiong, Boya Zhang, Xiao Yuan

**Affiliations:** 1 Guangdong Power Grid, Guangzhou 510080, China; firehuo_0403@126.com (L.W.); kevin0319kl@aliyun.com (S.H.); 2 China Southern Power Grid Co., Ltd., Guangzhou 510080, China; zhipeter@126.com; 3 State Key Laboratory of Electrical Insulation and Power Equipment, Xi’an Jiaotong University, Xi’an 710049, China; xiaoyuan0207@163.com

**Keywords:** density functional theory, first-principles calculation, charge injection barrier, HVDC

## Abstract

Comprehending charge injection at the metal/epoxy interface is essential for designing and applying high-voltage electrical equipment. This study investigates surface charge accumulation in insulators used in high-voltage direct current (HVDC) gas-insulated switchgear (GIS), with a specific focus on the charge injection behavior at the metal/epoxy interface employing first-principles calculations. In this paper, two amine curing agents were selected to construct interface models of a Cu(111) slab and epoxy resin, with repeating fragments representing the crosslinked structure of the resin. Key parameters, including injection barriers, charge transfer, and vacuum energy level shifts (Δ), were evaluated. Notably, molecular structures containing -C_2_F_6_ bonds exhibited higher electron and hole injection barriers compared to those with -CH_2_. Specifically, DDM induces reduced interfacial charge injection barriers and enhanced charge transport capabilities attributed to its low electronegativity and compact spatial configuration, whereas 6FDAM yields elevated barrier heights stemming from its strong electronegative character. The reliability of these findings was further validated through macroscopic charge injection experiments. The above study holds certain referential value for the development and application of high-voltage DC GIS equipment.

## 1. Introduction

The transmission of electrical energy has long been a critical challenge in power systems, and advancements in transmission technology are of paramount importance to the economic and social development of nations. Currently, high-voltage direct current (HVDC) transmission technology plays a pivotal role in enabling long-distance, large-capacity power transmission and facilitating the interconnection of power grids on a global scale. This technology not only facilitates the clean and efficient utilization of diverse energy sources, it also enables the optimal allocation of energy resources across broader geographical regions, thereby significantly advancing the development of electric power systems worldwide [1]. High-voltage DC gas-insulated switchgear (GIS), a critical component in DC power networks, exhibits high reliability, large transmission capacity, and minimal susceptibility to external environmental factors. Compared to open switchgear, it reduces volume by over 70%, significantly decreasing the footprint of both offshore and onshore converter stations, and holds vast potential for applications in DC power transmission.

Polymers have been widely employed as insulators within electrical applications. This is attributable to their cost-effectiveness, flexibility, excellent insulating characteristics, and chemical and thermal stability, as well as their ease of processing [2,3]. Nevertheless, under high-voltage conditions, typical polymers like polyethylene and polypropylene, along with rubber-like polymers, are prone to the accumulation of internal space charges, which has the potential to cause the dielectric breakdown of the insulating layer [4,5].

One of the most critical challenges throughout the long-term running of DC GIS is the accumulation of surface charges on insulators. Extensive experimental studies have demonstrated that surface charge accumulation under DC voltage is a significant factor contributing to the reduction in flashover voltage along the insulator surface [6,7,8,9]. In AC GIS, the polarity of the voltage and the direction of the electric field alternate periodically, making it difficult for charged particles to migrate continuously to the gas–solid interface and thus preventing significant charge accumulation. In the design and optimization of insulators for AC GIS, the steady-state AC electric field distribution is commonly used as the basis, without considering the effects of charge accumulation. However, the mechanisms of charge accumulation and failure in DC equipment differ fundamentally from those in AC equipment. The internal electric field follows a resistive distribution, rendering existing structural designs and materials inadequate for high-voltage DC applications. Furthermore, as DC GIS insulators are exposed to a unipolar electric field over extended periods, charges migrate steadily in the same direction under the influence of the DC electric field, leading to significant charge accumulation and limited dissipation. When the accumulated charge reaches a critical threshold, it distorts the local electric field at the gas–solid interface, potentially inducing surface flashover [10]. Moreover, under complex operating conditions involving large temperature gradients, polarity reversal, and DC superimposed impulses, the likelihood of insulator surface flashover increases significantly.

Numerous studies have been conducted globally to investigate the charge accumulation mechanisms at the gas–solid interface of tub insulators under DC conditions. It is widely accepted that surface charge accumulation primarily occurs through three mechanisms: (1) gas-side conduction driven by the electric field at the insulator interface; (2) solid-side conduction prompted by the electric field at the insulator interface; and (3) surface conduction along the insulator. Solid-side conduction arises from the long-term migration of space charges within the polymer material [11]. These migrating charges primarily consist of electrons and holes injected from the metal electrodes, as well as charges promoted from the polymer’s valence band to its conduction band [12]. Charge injection at the metal/polymer interface is a critical area of research in materials science and electrical engineering, as it directly influences the insulating properties and reliability of devices. In electrical components like capacitors and cables, charge injection at the metal/polymer interface is known to cause gradual polymer deterioration, ultimately resulting in the breakdown of the embedded dielectric layer [13,14]. As a key regulatory unit in the crosslinked network of epoxy resins, the molecular structure of curing agents directly determines the thermal stability, dielectric properties, and mechanical strength of the cured products. Optimizing the structure of curing agents through molecular design can effectively balance the high-temperature insulating performance and processing applicability of epoxy resins, thereby meeting the stringent requirements of advanced power equipment for material reliability under extreme working conditions [15]. Furthermore, interfaces between electrodes, semiconductors, and insulating materials, as well as interfaces between different insulating material structures, significantly influence charge injection and accumulation behavior in insulating materials [5,16]. However, the charge injection behavior at interfaces involving metal electrodes and epoxy resins with varying molecular structures remains understudied in the context of DC GIS.

Given the operational risks induced by surface charge accumulation on DC GIS insulators and the research gap regarding charge injection behavior at the interface between epoxy resins with varied molecular structures and metal electrodes, this study aims to clarify the charge injection mechanisms at such interfaces. It seeks to reveal how curing agent molecular structures regulate interfacial charge injection barriers, charge migration characteristics, and surface charge accumulation behavior, thereby addressing the key scientific issue of ambiguous correlations between interfacial charge injection behavior and molecular structures of epoxy insulators in DC GIS. The findings are expected to provide theoretical guidance for the molecular design and performance optimization of high-performance epoxy insulating materials for DC GIS, lay the foundation for developing new insulating materials with low charge accumulation properties, and further enhance the long-term operational reliability of DC GIS equipment. This holds significant practical value for advancing the safe and efficient development of high-voltage direct current transmission technology.

First-principles calculations offer a robust and effective approach for investigating metal/epoxy interfaces. Previous studies on interfacial charge injection have focused on characteristics such as ionization energy, electron affinity, and charge injection barriers [13,17], and thus the present study also centers on these features. This study investigates the influence of varying bridge bonding groups in amine curing agents on the charge injection properties at metal/epoxy interfaces. Metallic copper was selected as the electrode material in this study, primarily attributed to its exceptional electrical conductivity and extensive engineering applications in electrical equipment. Molecular fragments of crosslinked epoxy resin (EP) were positioned on the Cu(111) crystal surface to construct a model of the metal/epoxy interface. Electron density differences, vacuum energy level shifts, and injection barriers at various interfaces were computed using first-principles calculations in CP2K. These findings provide valuable insights for the study of HVDC GIS.

## 2. Models

### 2.1. Cu Slab Model

After obtaining the unit cell structure of metallic copper from the FindIt database, the (111) crystal plane, which exhibits minimal energy, was selected for constructing the crystal surface model [18]. Subsequently, the unit cell was cleaved to generate the desired crystal surfaces. To determine the optimal number of layers in the metal structure, the surface energy (*γ*) was employed as an evaluation criterion, calculated as follows [19]:

(1) $$\gamma =\frac{\left(E_{\mathrm{slab}}-nE_{\mathrm{bulk}}\right)}{2A}$$

**Figure 1:**
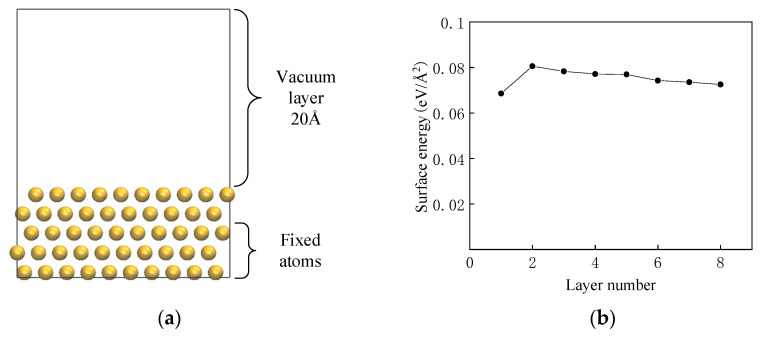
(**a**) Cu slab model (**b**) surface energy as a function of the layer number.

**Figure 2:**
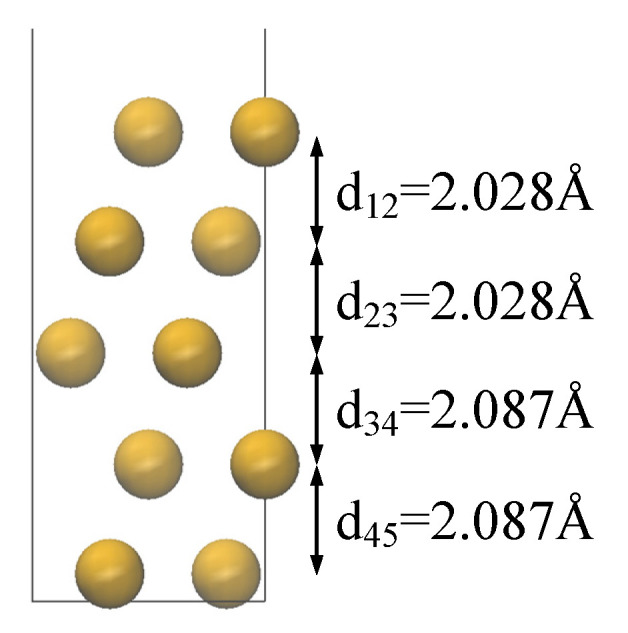
The layer distance of Cu slab; d_ij_ represents the distance between the ith layer and jth layer.

Here, *E*_slab_ represents the total energy of the metal surface model calculated via density functional theory, *E*_bulk_ denotes the energy per atom in the bulk structure, n is the ratio of the number of atoms in the metal surface model (*N*_slab_) to the number of atoms in the bulk metal cell (*N*_bulk_), defined as *n* = *N*_slab_/*N*_bulk_, and *A* is the surface area. As depicted in Figure 1b, when the number of layers reaches five, the surface energy variation stabilizes at less than 0.001 eV/Å^2^, converging to 0.77 eV/Å^2^ (1.45 J/m^2^), which is consistent with the experimental value of 1.78 J/m^2^ [20]. As illustrated in Figure 1a, a vacuum layer thickness of 20 Å was employed to eliminate interlayer interactions. The constructed supercells, with dimensions of 22.1376 Å × 10.2249 Å × 28.3486 Å, feature a (5 × 4) periodic structure and comprise 200 copper atoms. Furthermore, as depicted in Figure 2, the layer spacing variation is less than 0.01 Å when the number of relaxation layers reaches two.

The construction of metal interfaces and surface calculations were conducted using first-principles calculation software CP2K 2024.1 [21], while additional calculations were carried out using the CASTEP module within Materials Studio 2017 [22]. Exchange-correlation effects were calculated using the generalized gradient approximation (GGA) in conjunction with the Perdew–Burke–Ernzerhof (PBE) functional [23,24]. To accurately describe van der Waals interactions, the Tkatchenko–Scheffler (TS) method [25] was employed for dispersion correction. During the optimization process, the bottom two layers were fixed. The optimized copper cell was cleaved along the (111) plane and extended into a 5 × 4 supercell comprising five atomic layers. A 20 Å thick vacuum layer was introduced to the copper surface. During geometry optimization, the convergence threshold for self-consistent field (SCF) iterations was established at 1 × 10^−6^ eV/atom. The cutoff energy for plane-wave basis was established at 500 eV, and Brillouin zone integrations were performed using the Monkhorst–Pack method [26]. The convergence thresholds were defined as follows: for total energy variation, 2 × 10^−5^ eV/atom; for maximum force, 0.05 eV/Å; for maximum stress, 0.1 GPa; and for maximum displacement, 0.002 Å. Energy cutoffs of 450 eV, 600 eV, and 0.002 Å were used to calculate the electronic properties of the metal unit cell, metal surface model, and metal/epoxy interface, respectively. The SCF convergence criterion was set to 1 × 10^−6^ eV/atom. The interfacial structure comprises more than 250 atoms.

### 2.2. Epoxy Model

**Figure 3:**
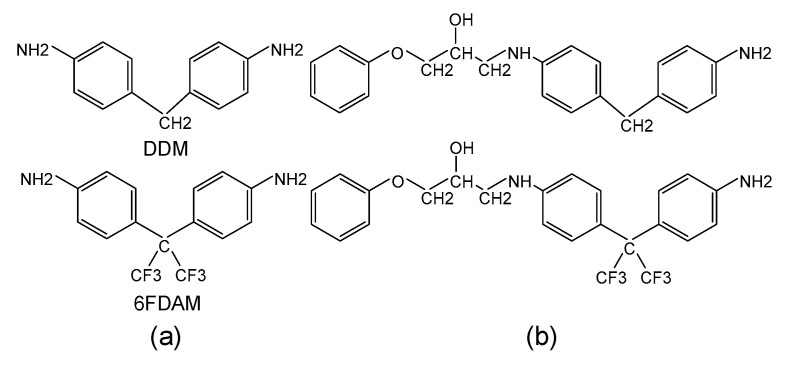
(**a**) The two aniline curing agents and the (**b**) corresponding epoxy molecular fragments used in this study.

The fragment structures of epoxy crosslinked systems provide a valuable approach for investigating charge injection barriers at the metal/epoxy (EP) interface [27,28]. The two epoxy resins investigated in this study were cured using an aniline-based curing agent, with the primary distinction being the bridge bonding groups -CH_2_ and -C_2_F_6_, as illustrated in Figure 3a. The epoxy molecular fragments resulting from reactions with the two selected curing agents are depicted in Figure 3b. The terminal ends of the sheared fragments were saturated through auto-hydrogenation to stabilize the chain structure.

### 2.3. Metal/Epoxy Interface Model

**Figure 4:**
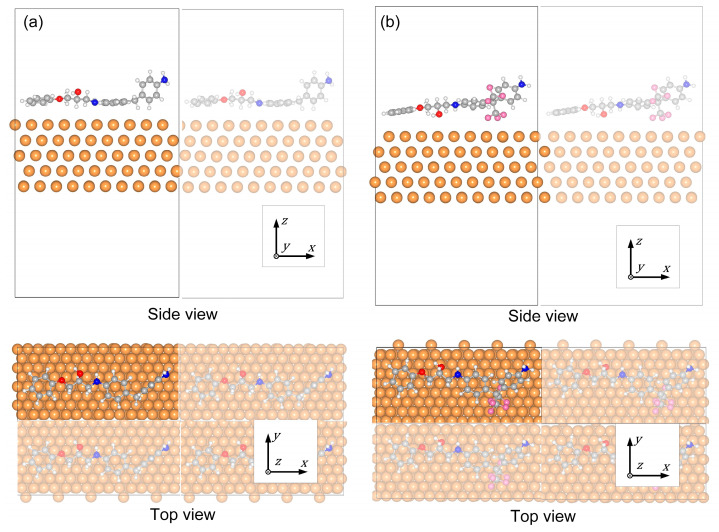
Side and top views of the structure of the interface model with (**a**) a DDM-EP structure and (**b**) a 6FDAM-EP.

The sheared fragments were positioned on the copper surface following the saturation of the chain segment structure through auto-hydrogenation. Figure 4a,b depict the optimized final structures of the Cu/DDM-EP and Cu/6FDAM-EP interface models following the binding of the fragments to the copper surface. The six-membered rings of the epoxy molecular fragments are oriented parallel to the copper surface, with an average distance of approximately 3.5–4 Å between the epoxy molecules and the metal surface, where the determination of this distance referred to the relevant research [29]. The sixmembered ring from the DDM-EP fragment is approximately parallel to the Cu surface. The -CF_3_ groups in the Cu(111)/6FDAM-EP are oriented toward the Cu surface. The black solid boxes indicate the unit cell boundaries, while the atoms of hydrogen, carbon, oxygen, fluorine, nitrogen, and copper are depicted in white, gray, red, pink, blue, and yellow, correspondingly.

To achieve an accurate representation of the metal/epoxy interface, the overall geometry of the model must be optimized. In this study, the interfacial model was optimized using density functional theory (DFT), with simulations performed using CP2K software, which is particularly effective for large-scale periodic systems.

### 2.4. DFT Simulation

Density functional theory (DFT) was employed for geometric optimization and electronic structure calculations of interfacial models, utilizing the Perdew–Burke–Ernzerhof (PBE) generalized gradient approximation as the exchange-correlation functional with DFT-D3(BJ) dispersion correction incorporated to properly describe van der Waals interactions. System optimization and single-point energy calculations were conducted at the PBE-D3(BJ)/DZVP-MOLOPT-SR-GTH computational level, with self-consistent field (SCF) calculations converging to a tolerance of 1 × 10^−6^ eV/atom. Geometric optimization convergence criteria included maximum atomic displacement within 3 × 10^−3^ Bohr and root-mean-square (RMS) atomic displacement within 0.0015 Bohr, while force convergence was achieved with maximum atomic force within 4.5 × 10^−4^ Bohr/Hartree and RMS atomic force within 0.0003 Bohr/Hartree.

Owing to the mismatch in Fermi energy levels at the interface, charge carriers migrate across the boundary. The accumulation of charge at the interface leads to the creation of a dipole moment and triggers a shift in the vacuum energy level (Δ). Typically, the electron injection barrier (*ϕ*_e_) and the hole injection barrier (*ϕ*_h_) can be represented as follows:

(2) $$\begin{array}{l} \phi_{e}=\psi_{m}-\mathrm{EA}+\Delta \\ \phi_{h}=-\psi_{m}+\mathrm{IP}-\Delta \end{array}$$

Here, *ψ*_m_ represents the metal work function, EA represents the electron affinity energy of the polymer molecule, IP denotes the ionization energy of the polymer, and Δ is derived from the variation in the work function of the metal surface model, specifically the difference between the model with and without the attached organic molecules [14,15]. The vertical electron affinity energy and ionization energy are computable by employing the ΔSCF approach,

(3) $$\begin{array}{l} \mathrm{EA}=E\left(N-1\right)-E\left(N\right)\\ \;\mathrm{IP}\;=E\left(N\right)-E\left(N+1\right) \end{array}$$

Here, *E*(*N*) represents the ground-state energy of a neutral molecule with N electrons in vacuum, and *E*(*N* − 1) corresponds to the ground-state energy of the molecule after losing one electron, forming a +1 valence ion. *E*(*N* + 1) denotes the ground-state energy of the molecule after gaining one electron, forming a −1 valence ion.

## 3. Result and Discussion

**Figure 5:**
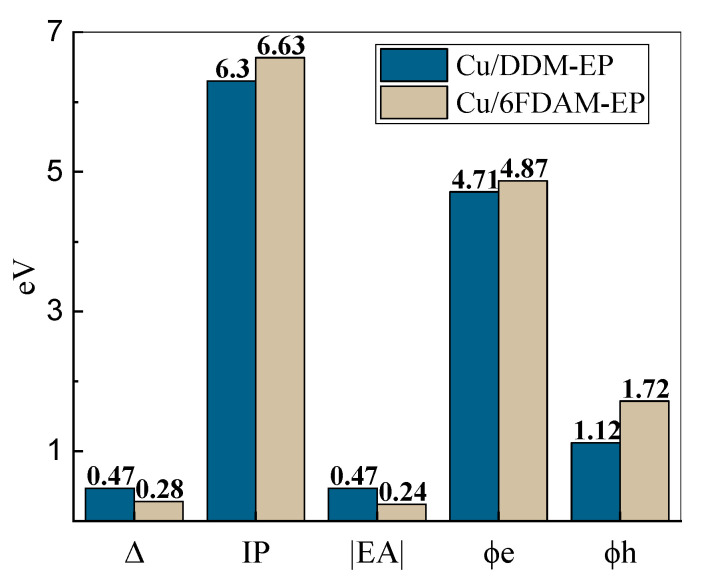
The computed charge injection characteristics of two interfaces.

The calculated charge injection parameters for the two interface configurations are summarized in Table 1 and illustrated in Figure 5.

N represents the total number of atoms in the model, d refers to the mean separation between the molecule and the metal surface, Δ denotes the shift in the vacuum energy level, IP stands for the ionization energy, EA is the electron affinity energy, *ϕ*_e_ is the electron injection barrier, and *ϕ*_h_ is the hole injection barrier.

The average distance d between the molecules and the metal surface is 3.73 Å for the Cu/DDM-EP interface and 3.79 Å for the Cu/6FDAM-EP interface. The shorter distance may enhance interfacial interactions, thereby influencing charge transfer behavior. The vacuum energy level shift Δ for Cu/DDM-EP is 0.468 eV, which is significantly higher than the 0.281 eV for Cu/6FDAM-EP. This indicates more pronounced energy changes and greater interfacial energy level modifications in Cu/DDM-EP, which may promote charge injection.

The ionization energy of DDM-EP is 6.30 eV, lower than the 6.63 eV of 6FDAM-EP, indicating that electrons in DDM-EP are more readily ionized. Additionally, the electron affinity of DDM-EP is −0.47 eV compared to −0.24 eV for 6FDAM-EP, suggesting that DDM-EP exhibits weaker electron affinity. These differences primarily arise from the group properties in the molecular structure, where the -C_2_F_6_ bridge bonding group in 6FDAM-EP plays a critical role. Compared to the -CH_2_ group in DDM-EP, the -C_2_F_6_ group exhibits stronger electronegativity, enabling it to effectively adsorb π electrons from the benzene ring in the curing agent and thereby reduce the nonlocal electron density. This effect not only elevates the ionization energy of 6FDAM-EP but also enhances its electron-attracting capability, resulting in a higher electron affinity energy, which is consistent with the strategy of modulating benzene ring electron density through strong electron-withdrawing groups, reported in relevant studies [30].

In addition, considering the electron injection barrier *ϕ*_e_ and hole injection barrier *ϕ*_h_, the values of *ϕ*_e_ and *ϕ*_h_ for Cu/6FDAM-EP are 4.87 eV and 1.72 eV, respectively. These values exceed the values for Cu/DDM-EP, which are 4.71 eV and 1.12 eV, respectively. This indicates that the charge injection barrier at the Cu/6FDAM-EP interface is larger, mainly attributed to the combined influence of its higher ionization energy and stronger electron affinity. Specifically, the higher ionization energy elevates the difficulty of electron injection. Simultaneously, the stronger electron affinity suppresses electron trapping. Consequently, a higher charge injection barrier is formed at the Cu/6FDAM-EP interface, which affects the charge transport characteristics, similar to how optimized molecular structures (e.g., MCDEA-cured EP) enhance interfacial barriers by regulating intermolecular stacking [30].

To gain a more in-depth understanding of the charge transfer and injection characteristics of the interfacial structures, the electron density difference Δ*ρ* among different interfacial structures was calculated as

(4) $$\Delta \rho =\rho_{\mathrm{Cu}-\mathrm{Epoxy}}\left(z\right)-\rho_{\mathrm{Cu}}\left(z\right)-\rho_{\mathrm{Epoxy}}\left(z\right)$$

where *ρ*_Cu-Epoxy_(z), *ρ*_Cu_(z), and *ρ*_Epoxy_(z) represent the average charge densities of the interface between Cu/EP, the surface of Cu, and the epoxy molecules along the *z*-axis, respectively.

**Figure 6:**
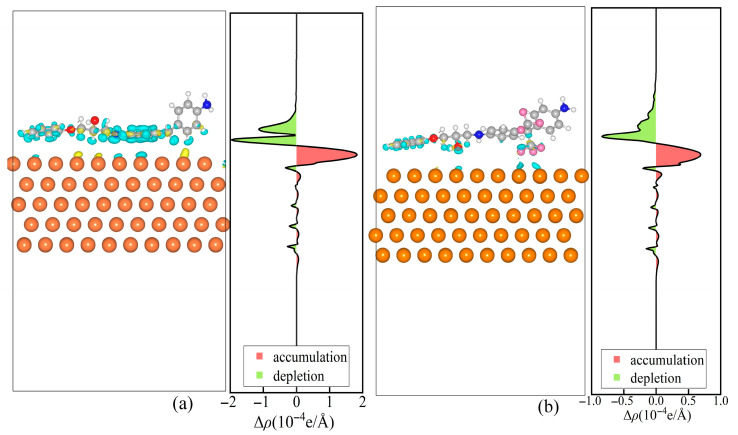
Electron density difference at the interface of (**a**) Cu(111)/DDM and (**b**) Cu(111)/6FDAM.

In this paper, the plane-averaged electron density difference is computed via Multiwfn 3.8 [31], and the electron density difference is visualized using VMD 1.9.3 [32]. Figure 6 depicts the electron density difference distributions Δ*ρ* of the Cu/DDM-EP and Cu/6FDAM-EP interfaces, along with the average electron density difference distribution in the direction perpendicular to the interface. The iso-surfaces of the electron density difference maps shown on the left side of Figure 6a,b have a value of 0.002 e/Å. The green color signifies an increase in electron density (electron distribution), while the blue color signifies a decrease in electron density (hole distribution). Electrons accumulate in the interface region (where Δ*ρ* > 0) and disperse in the DDM-EP and 6FDAM-EP regions (where Δ*ρ* < 0). Owing to the negligible change in Δ*ρ* within the metal Cu, the interfacial electrons predominantly originate from the DDM-EP and 6FDAM-EP fragment structures.

The maximum value of the average electron density difference is 2 × 10^−4^ eV/Å on the DDM-EP side and 0.8 × 10^−4^ eV/Å on the 6FDAM-EP side. Moreover, both the positive and negative values of Δ*ρ* are greater for the Cu/DDM-EP interface compared to the Cu/6FDAM-EP interface. This is because the DDM-EP molecule is closer to the Cu(111) surface than the 6FDAM-EP molecule. Additionally, the two benzene rings of the DDM-EP molecule are oriented parallel to the Cu(111) surface, and the interaction between the π-bonds within the benzene rings and the Cu atoms facilitates the charge transfer. Meanwhile, the simulation calculations presented in Table 1 reveal that both the electron injection barrier and the hole injection barrier of the Cu/6FDAM-EP model exceed those in the Cu/DDM-EP model. The injection barrier represents the energy that the charge within the epoxide small molecule must overcome to reach the metal surface, and this is manifested in the electron density difference curves. Specifically, both the positive and negative values of Δ*ρ* for the Cu/DDM-EP interface are greater than those for the Cu/6FDAM-EP interface.

Figure 6a reveals that the average electron density difference curve of the DDM molecule displays a “bimodal pattern”. This is due to the fact that the two benzene rings in the DDM molecule are oriented parallel to the metal surface. The strong interaction between the π-bonds in the benzene rings and the copper atoms drives the electrons to migrate towards the copper surface. Simultaneously, the electron repulsion leads to the accumulation of electrons above the benzene rings, thereby giving rise to the “bimodal pattern”. This structural characteristic facilitates the interfacial charge transfer between DDM-EP and the copper surface.

**Figure 7:**
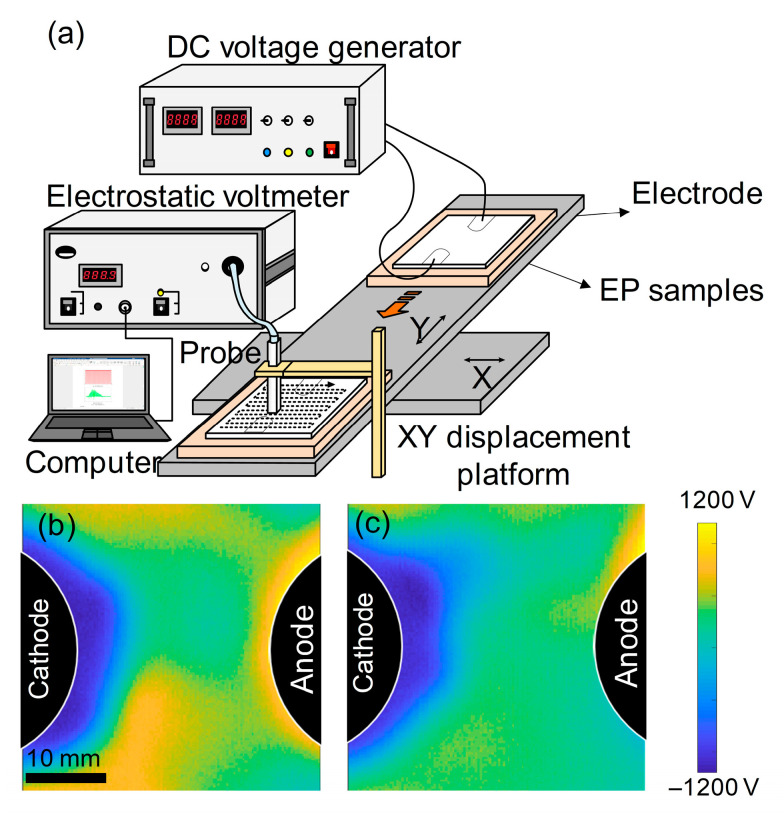
(**a**) Schematic of macroscopic charge injection and surface potential scanning. Surface potential distribution for EPs cured with (**b**) DDM and (**c**) 6FDAM-DBP.

To validate the aforementioned conclusions, the corresponding epoxy resin samples were fabricated, and macroscopic charge injection experiments were conducted. As schematically illustrated in Figure 7a, surface potential mapping was conducted with a Trek Model 341B electrostatic voltmeter coupled to a 3455ET micro-spacing probe (Trek, Inc., Lockport, NY, USA). The experimental samples comprised 35 mm × 38 mm × 0.3 mm epoxy sheets, while prototype validation samples featured 100 mm diameter circular geometries with identical 0.3 mm thickness. Charge injection was implemented through copper electrodes driven by a Keithley Model 6517B high-voltage DC source (Keithley Instruments, Inc., Solon, OH, USA). A computer-controlled XY translation stage enabled automated surface scanning, generating high-resolution potential distribution profiles across the specimen surfaces under applied DC fields. As depicted in Figure 7a, the potential of the sample was measured using an electrostatic probe following the application of a DC electric field. Based on the surface potential distributions presented in Figure 7b,c, it is evident that the DDM-cured epoxy resin established a broader charge accumulation region adjacent to the metal/polymer interface. This observation indicates that the DDM-cured epoxy resin possesses more pronounced interfacial charge injection and diffusion abilities. Moreover, the benzene rings of the DDM-cured epoxy resin are oriented parallel to the copper surface, which intensifies the interactions between the π-bonds and the metal, thereby facilitating the interfacial charge transfer.

In contrast, the 6FDAM-cured epoxy resin reduced the effective contact area between its molecules and the metal. This is attributed to the strong electronegativity of the -C_2_F_6_ group and its larger spatial volume. Additionally, the 6FDAM-cured epoxy resin trapped some of the carriers and hindered the charge injection and migration processes, leading to higher injection barriers and narrower charge distribution regions. The experimental results further verified that the 6FDAM-cured epoxy resin has a higher interfacial injection barrier. Collectively, these findings highlight distinct performance trade-offs between the two systems. DDM-EP exhibits enhanced charge injection and diffusion capabilities but may pose risks of excessive long-term charge accumulation under prolonged DC stress. Conversely, 6FDAM-EP benefits from higher injection barriers that effectively suppress charge ingress—advantageous for improving insulation stability—though its reduced interfacial contact area, arising from the steric hindrance and electronegativity of -C_2_F_6_, might compromise interfacial bonding strength in practical engineering scenarios.

The formation of a higher interfacial barrier in 6FDAM-EP may be attributed to the following two factors. Firstly, the -C_2_F_6_ bridge bonding group in 6FDAM exhibits a stronger electronegativity compared to the -CH_2_ group. This characteristic enables it to adsorb π-electrons on the benzene ring within the curing agent, consequently decreasing the nonlocal electron density. Simultaneously, it functions as a carrier trap, effectively impeding the charge injection and migration processes at the metal/epoxy interface. Secondly, due to the fact that the -C_2_F_6_ bridge bonding group has a larger spatial volume than the -CH_2_ group, it can decrease the effective contact area both between molecular chains and at the interface between molecular chains and the metal. This, in turn, weakens the metal/epoxy interaction and raises the electron/hole injection barrier at the interface.

## 4. Conclusions

In this study, a comprehensive examination of the metal/epoxy interface characteristics is executed by means of first-principles calculations, with a focus on analyzing the influence of bridge bonding group differences in amine curing agents regarding the charge injection characteristics at the metal/epoxy interface. For simulation modeling, a crystalline surface model of metallic copper was accurately constructed, the number of metal layers was determined based on surface energy, and parameters such as the vacuum layer thickness were optimized. Specific software and computational methods were employed to construct the metal interface and perform related calculations. For epoxy modeling, two epoxy resins cured with distinct aniline-based curing agents were utilized. In constructing the metal/epoxy interface model, the treated molecular fragments were positioned on the copper surface, and geometry optimization was conducted via density functional theory (DFT) and CP2K software, with strict convergence criteria applied to ensure computational accuracy and reliability. Systematic theoretical analyses and preliminary calculations reveal that the characteristics of the charge injection barrier are more strongly associated with the electron affinity and ionization energy inherent to the epoxy compounds themselves. In contrast, the vacuum level shift induced by different metals is negligible. Consequently, the research conclusions derived from copper-based studies can be reasonably extended to other commonly used metals, thereby ensuring the universality of the research findings.

The simulation results reveal significant differences in the charge injection properties of the two interfacial configurations, Cu/DDM-EP and Cu/6FDAM-EP. The distance between the polymer molecules and the metal surface is 3.73 Å for Cu/DDM-EP and 3.79 Å for Cu/6FDAM-EP, with both values influencing charge transfer to varying degrees, despite the small difference. The vacuum energy level shift is 0.468 eV for Cu/DDM-EP, higher than the 0.281 eV for Cu/6FDAM-EP, indicating more pronounced energy changes in Cu/DDM-EP. Analysis of ionization energy reveals that Cu/DDM-EP (6.30 eV) has a lower ionization energy than Cu/6FDAM-EP (6.63 eV), suggesting that Cu/DDM-EP is more readily ionized. The electron affinity of Cu/DDM-EP (−0.47 eV) is lower than that of Cu/6FDAM-EP (−0.24 eV), a difference attributed to the distinct bridge bonding groups. The -C_2_F_6_ group contributes to the higher ionization energy and enhanced electron affinity of Cu/6FDAM-EP. As a consequence, it gives rise to higher electron and hole injection barriers, which ultimately leads to the emergence of greater electron and hole injection barriers. Specifically, the electron injection barrier for Cu/6FDAM-EP is 4.87 eV and the hole injection barrier is 1.72 eV. In contrast, for Cu/DDM-EP, these values are 4.71 eV for the electron injection barrier and 1.12 eV for the hole injection barrier. Electron density difference calculations reveal electron aggregation in the interface region and dissipation in the DDM-EP and 6FDAM-EP regions. The positive and negative electron density differences (Δ*ρ*) for Cu/DDM-EP are greater than those for Cu/6FDAM-EP, primarily due to the unique structural relationship between DDM-EP and the Cu(111) surface, which enhances charge transfer. The electron density difference curves of DDM-EP molecules exhibit a bimodal distribution, facilitating the injection of interfacial electrons and holes. Macroscopic charge injection experiments further validate these findings, demonstrating that DDM-EP forms a broad charge accumulation region near the metal/polymer interface, whereas 6FDAM-EP exhibits a higher interfacial barrier. This elevated potential barrier is primarily due to the strong electronegativity and larger spatial volume of the -C_2_F_6_ bridge bonding group. The former acts as a carrier trap by adsorbing electrons, thereby inhibiting charge injection and migration, while the latter reduces the contact area, reducing the metal/epoxy interaction strength, thereby enhancing the electron and hole injection barriers at the interface.

In summary, this study delves into the mechanisms related to how charges are injected at the interface between metal and epoxy in high-voltage DC GIS, providing theoretical insights and data to support the enhancement of insulation performance and structural optimization of DC GIS equipment. Following the presentation of research results, it systematically clarifies the charge injection mechanisms, specifically revealing the link between material properties and injection barriers. Although further investigation is required to address remaining challenges, this work identifies key challenges such as the combined impact of various environmental factors on interfacial charge behavior, which call for targeted follow-up studies. It offers valuable insights and inspiration for research on high-voltage direct current (HVDC) equipment, particularly in improving operational stability and reliability.

## 5. Perspectives

Subsequent research will extend the structure–performance relationships established herein—particularly the distinct impacts of -C_2_F_6_ and -CH_2_ groups on charge injection barriers—by examining diverse curing agent functional moieties to develop a framework linking molecular structures to interfacial charge behavior. It will also investigate charge dynamics under GIS-specific complex conditions to elucidate how operational factors regulate the observed barrier effects, refine nonlinear interface modeling via tools like fractional calculus to capture unaddressed time-dependent charge accumulation, and validate optimized formulations in full-scale GIS insulator prototypes, thereby translating molecular-level insights into engineering applications to enhance HVDC system reliability.

## Figures and Tables

**Table 1:** The computed charge injection characteristics of two interfaces.

Model	N	D (Å)	Δ (eV)	IP (eV)	EA (eV)	*ϕ*_e_ (eV)	*ϕ*_h_ (eV)
Cu/DDM	250	3.73	0.468	6.30	−0.47	4.71	1.12
Cu/6FDAM	256	3.79	0.281	6.63	−0.24	4.87	1.72

## Data Availability

The original contributions presented in this study are included in the article. Further inquiries can be directed to the corresponding author.
